# Three-Dimensional Measurements of Sphenoid Sinus Size by Sex in a Korean Population: An Exploratory Study

**DOI:** 10.3390/diagnostics14242888

**Published:** 2024-12-23

**Authors:** Jeong-Hyun Lee, Jong-Tae Park

**Affiliations:** 1 Department of Oral Anatomy, Dankook Institute for Future Science and Emerging Convergence, Dental College, Dan-Kook University, Cheonan 31116, Republic of Korea; 911105jh@dankook.ac.kr; 2 Department of Bio Health Convergency Open Sharing System, Dan-Kook University, Cheonan 31116, Republic of Korea

**Keywords:** sphenoid sinus, 3D modeling, sinus, cone-beam computed tomography, sex differences

## Abstract

**Background/Objectives:** This study aims to investigate the three-dimensional morphological differences of the sphenoid sinus according to sex in the Korean adult population and conduct an exploratory study based on the findings. The sphenoid sinus, located deep within the skull, plays a crucial role in forensic identification due to its relative protection from external damage and its unique anatomical characteristics. **Methods:** Using cone-beam computed tomography (CBCT) data from 120 patients (60 males and 60 females) aged 20–29, the sphenoid sinus was visualized and measured in three dimensions using Mimics software (version 22.0). Measurements included the volume of the sphenoid sinus, as well as its dimensions in the X, Y, and Z axes. The measured data were analyzed using SPSS (version 23.0) with a *t*-test and linear regression analysis. **Results:** The results showed that the sphenoid sinus volume was significantly larger in males compared to females (*p* < 0.05), with an average male sinus volume of 16,957.9 mm^3^ and a female volume of 13,517.7 mm^3^. Additionally, the X-width, Y-width, and Z-width were all larger in males, with significant differences (*p* < 0.001) across all dimensions. Further regression analysis revealed that the volume of the sphenoid sinus was primarily influenced by the Z-axis height (measured from the coronal view) and the Y-axis width (measured from the sagittal view), while the X-axis width had a negligible effect on the overall volume. **Conclusions:** These findings suggest that sex-specific differences in the sphenoid sinus may provide important insights for clinical diagnoses and forensic personal identification. This study highlights the need for further research on different age groups and ethnic populations to enhance the understanding of anatomical variations in the sphenoid sinus and their potential applications in both medical and forensic fields.

## 1. Introduction

The sphenoid sinus is an air-filled cavity located deep within the skull, playing a significant role in clinical and forensic sciences [1,2]. Positioned near critical structures such as the brain, optic nerve, and pituitary gland, it holds immense importance in medical diagnoses and surgical procedures [1,3]. However, studies on the sphenoid sinus have predominantly focused on Western populations [1,2,3,4,5,6,7,8,9,10,11,12,13,14,15,16,17], leaving a gap in understanding its anatomical variations among East Asian populations, including Koreans. Given the known anatomical differences across ethnic groups, this lack of data is particularly concerning. Comprehensive 3D-imaging-based analyses of the sphenoid sinus in Koreans are rare, necessitating targeted research.

Research on paranasal sinuses has demonstrated their unique and individualized morphologies, which are useful for personal identification [4,5,6,7]. Extensive studies have been conducted on the frontal and maxillary sinuses [4,5,6,7]; however, their susceptibility to external damage limits their forensic utility [7]. In contrast, the sphenoid sinus, located deep within the nasal cavity, is less vulnerable to trauma, offering greater reliability for identification purposes [7]. Technological advancements in medical imaging have facilitated new research in forensic anthropology [18]. Radiography is commonly used, and the methods for obtaining and visualizing images have continuously improved [19]. CT, known for its accuracy and reliability in analyzing skeletal elements, has seen increased usage in studies visualizing and analyzing data [20,21,22]. This advancement aids in identifying new anatomical structures and supports research and data collection [23]. Currently, three-dimensional (3D) studies on the sphenoid sinus, which is located in a relatively deep area, are underway [1,2,3,4,5,6,7,8,9,10,11,12,13,14,15,16,17]. However, most of these studies do not use specialized 3D software, necessitating research utilizing such programs.

This study aims to fill this gap by providing valuable insights into the anatomical variations of the sphenoid sinus in Koreans, thereby enhancing its clinical and forensic applications.

Advancements in technologies such as Mimics software have enabled precise 3D visualization and measurement of anatomical structures. This software converts two-dimensional (2D) medical imaging data into accurate 3D models, overcoming the limitations of traditional methods and offering deeper insights into the morphology of the sphenoid sinus. This study leverages such technology to evaluate sex-based differences in the size and volume of the sphenoid sinus, aiming to provide critical data to enhance its clinical and forensic applications.

To achieve this, this study sets forth the following objectives and hypotheses. The primary objective is to investigate the three-dimensional morphological differences in the sphenoid sinus between males and females within a Korean adult population aged 20–29 years. This exploratory study seeks to answer the following questions: First, is there a significant difference in the size and volume of the sphenoid sinus between male and female subjects? Second, to what extent do the dimensions measured along the X-axis (width in coronal view), Y-axis (width in sagittal view), and Z-axis (height in coronal view) influence the overall volume of the sphenoid sinus?

Based on these questions, the study proposes two hypotheses: Hypothesis 1: There is no significant difference in the size and volume of the sphenoid sinus between male and female subjects. Hypothesis 2: The overall volume of the sphenoid sinus is not significantly influenced by the dimensions measured along the X, Y, and Z axes.

These hypotheses are grounded in existing literature, which suggests potential sexual dimorphism in craniofacial structures and variability in sinus expansion during growth. Through these objectives and hypotheses, this study aims to contribute to the body of knowledge on the sphenoid sinus. The primary objective of this study is to generate valuable data for anatomical and forensic analyses of the sphenoid sinuses in Koreans by employing advanced 3D modeling techniques to assess their size and volume. Using these findings as a foundation, an exploratory study was subsequently conducted.

## 2. Materials and Methods

### 2.1. Subjects

The subjects of this study consisted of 120 individuals (60 males and 60 females) aged 20–29 who visited Dankook University Dental Hospital. The classification of participants’ sex was based on self-reported information provided at the time of their visit. The subjects had no missing teeth, systemic asymmetry, or diseases. Ethical approval for the retrospective analysis was obtained from the Institutional Review Board (IRB) of Dankook University (Approval No.: DKUDH IRB 2020-01-007). The participants were not specifically recruited for this study but were selected among patients who met the inclusion criteria during their visit to Dankook University Dental Hospital.

The sample size was determined using the G-Power 3.1 program (HHU, Düsseldorf, Germany). The sample size calculation was based on the following parameters: Test type: *t*-tests, Statistical test: mean difference between two independent means (two groups), Power analysis type: a priori (calculating the required sample size given α, power, and effect size). The input parameters were set as follows: Tails: two, Effect size (d): 0.8, α error probability: 0.05, Power (1 − β error probability): 0.95, Allocation ratio (N2/N1): 1. As a result, the required sample size was calculated to be 42 participants per group, totaling 84 participants. An additional 36 participants were included, resulting in a total of 120 participants in this study.

Furthermore, in this study, the size of the sphenoid sinus in patients in their 20s was used as the measurement standard to minimize normal size variations and anatomical asymmetry differences after reaching adulthood.

### 2.2. Methods

#### 2.2.1. CBCT Data Acquisition

The data for this study were acquired with the following measures to ensure accuracy and minimize errors. During CBCT imaging, all patients maintained a neutral posture to increase consistency. To further reduce errors, the Frankfurt horizontal (FH) plane was positioned perpendicular to the floor, and the computed tomography scanner (Alphard 3030, Asahi, Kyoto, Japan) was aligned with the sagittal midline of the face during imaging. The images were obtained under the following conditions: gantry angle 0°, 120 kV, auto mA, Slice Increment 0.39 mm, Slice Thickness 0.39 mm, Slice Pitch 3, Scanning Time 4 s, matrix 512 px × 512 px. All images were saved in DICOM format.

#### 2.2.2. Mathematical Techniques and Modeling in Mimics Program

The Mimics (version 22.0, Materialise, Leuven, Belgium) program applies various mathematical techniques and modeling to convert 2D scan data obtained from CT or MRI into 3D models.

1. Mathematical Analysis

• Segmentation

Image segmentation is the process of identifying and extracting specific anatomical structures from scan data. The Mimics program primarily uses thresholds to separate regions of interest based on Hounsfield Unit (HU) values. Additionally, a region-growing algorithm is used to manually specify regions of interest more accurately.

• Masking

To isolate the desired anatomical structure in the Mimics program, the pixels of the region are binarized to create a mask. This allows extraction of only the part that includes the desired tissue.

• Interpolation

The Mimics program uses methods such as linear interpolation or spline interpolation to fill in data between adjacent 2D slices. This reduces the gap between slices and helps create a more continuous 3D model.

• Triangular Mesh Generation

In the final stage of 3D modeling, segmented data are converted into a triangular mesh to define the surface. The marching cubes algorithm is used for surface extraction, creating a 3D mesh from the data. The generated mesh consists of thousands of triangles, allowing the visualization of complex anatomical surfaces.

2. Mathematical Modeling Analysis

The Mimics program utilizes mathematical modeling to analyze data and provide patient-specific solutions using various mathematical techniques.

• 3D Transformation and Coordinate System

To visualize the model or analyze it from different angles, the Mimics program uses matrix transformation. This process includes rotation, translation, and scaling, allowing accurate adjustment of the model’s position and orientation in 3D space.

• Curvature and Surface Analysis

The Mimics program calculates the surface curvature of complex anatomical structures to analyze irregularities. Curvature analysis is useful for measuring morphological characteristics, such as the curved contours of structures like the sphenoid sinus.

• Volume and Distance Calculation

It is possible to calculate the volume of segmented structures and the distance between two structures.

#### 2.2.3. 3D Modeling

The provided DICOM files were modeled in 3D using the Mimics 3D program. The sphenoid sinus was extracted using the following steps.

1. Importing CBCT Data

**Figure 1:**
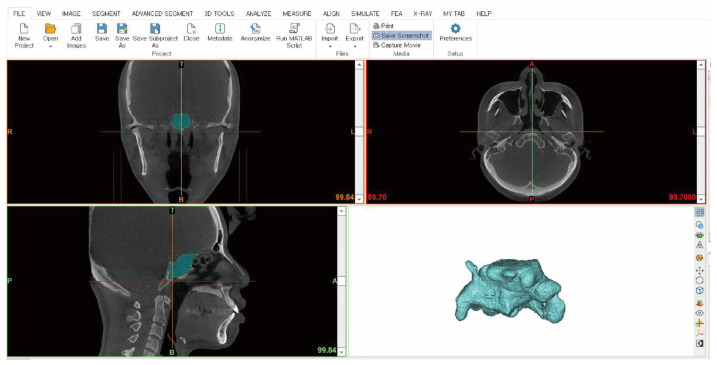
Importing CBCT data.

The provided CBCT data were loaded into a new project, and image alignment was performed. During image alignment, the obtained scan data are automatically aligned with the coordinate plane, and images in the coronal, sagittal, and frontal views are generated through rotation and translation transformations according to anatomical standards (Figure 1).

2. Thresholding

**Figure 2:**
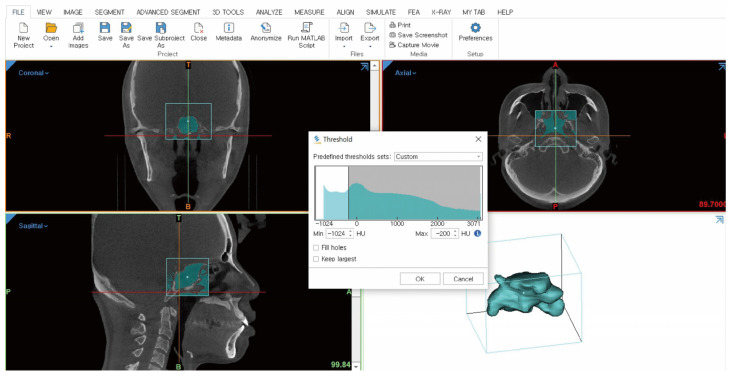
Thresholding.

To perform segmentation, the location of the sphenoid sinus was identified from the loaded CBCT. After locating the sphenoid sinus, the “New Mask” option in the segment tab was clicked to set the threshold. The threshold was set manually by entering HU values suitable for extracting the sphenoid sinus, with a minimum of −1024 and a maximum of −260. The “Crop Mask” function was used to manually define the detected sphenoid sinus area, and a mask was created. During mask creation, interpolation and the marching cubes algorithm were automatically applied. Interpolation fills in the gaps between slices to create a 3D shape from 2D data. Additionally, the marching cubes algorithm generates a triangular mesh to form the 3D surface. These two functions were used to smoothly represent the curves and slopes of the sphenoid sinus (Figure 2).

3. Manual Correction

**Figure 3:**
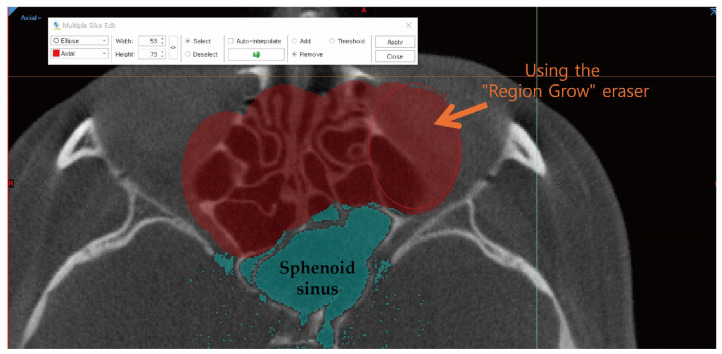
Manual correction. Red + is using the Multiple Slice Edit function to remove noise; The blue part represents the mask of the sphenoid sinus.

To completely remove unnecessary bones and noise surrounding the 3D-generated sphenoid sinus, the “Multiple Slice Edit” and “Region Grow” functions were used. “Multiple Slice Edit” was applied to remove adjacent structures such as unnecessary bones and noise by marking and deleting them on every fifth slice in the axial view of the 2D image data. Any additional noise was manually removed using the “Region Grow” function (Figure 3).

4. STL File Conversion

**Figure 4:**
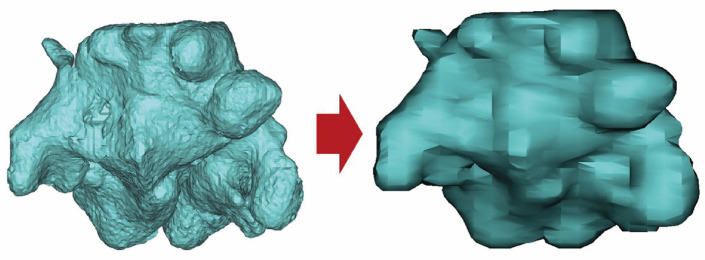
STL file conversion.

The 3D sphenoid sinus created as a mask was converted into an STL file to improve model precision and facilitate further analysis. The “Calculate Part” function was used to automatically convert the mask to an STL file (Figure 4).

#### 2.2.4. Method for Separating Adjacent Structures of the Sphenoid Sinus

In this study, the following methods were used to distinguish and separate the sphenoid sinus from adjacent structures, such as aerated septal cells.

1. Threshold Setting Using HU Values

Mimics software utilizes HU values to segment structures. In this study, a specific HU range was applied to isolate the sphenoid sinus from other tissues. The HU values for all data were consistently set between Min −1024 and Max −260. This threshold range effectively identified the air-filled sphenoid sinus while excluding structures like soft tissues.

2. Manual Editing and Cropping

After generating an initial mask using the threshold function, the software allowed researchers to refine the segmentation by manually removing unnecessary structures. The “Crop Mask” function was used to specifically define the sphenoid sinus, ensuring that adjacent aerated structures, such as septal cells, were excluded. Additionally, the “Multiple Slice Edit” function was employed to review and remove any remaining bones or undesired structures.

3. Cross-sectional Validation

The sphenoid sinus was visualized in three perspectives: coronal, sagittal, and frontal views. These views were cross-referenced to accurately distinguish the sphenoid sinus from adjacent aerated structures, such as septal bubbles. By combining HU-based thresholding, manual segmentation adjustments, and multi-view validation, this method effectively distinguished the sphenoid sinus from adjacent aerated structures. This approach minimized the inclusion of undesired regions in the analysis.

#### 2.2.5. Measurement Items

To ensure the accuracy of all measurements and minimize errors, the Frankfurt horizontal line was adjusted to a horizontal position before proceeding with the measurements.

1. Setting the Reference Point

The Analyze Point function was used to set the highest point of the sphenoid sinus as a reference point. This reference point minimizes errors during repeated measurements.

2. Length Measurement

The “Distance” function was used to measure the length of the sphenoid sinus along specific axes (X, Y, Z).

3. Volume Calculation

The volume of the 3D sphenoid sinus was measured based on the generated mesh surface. Once the 3D STL file is created, the volume value is automatically calculated, and the data can be obtained from the Properties.

4. Repeated Measurements and Reliability Verification

Two researchers performed each measurement twice, and the average value was calculated. The reliability of the measurements was assessed (Cronbach’s α = 0.709), and statistical analysis was conducted after reliability verification.

5. Measurement Parameters

**Figure 5:**
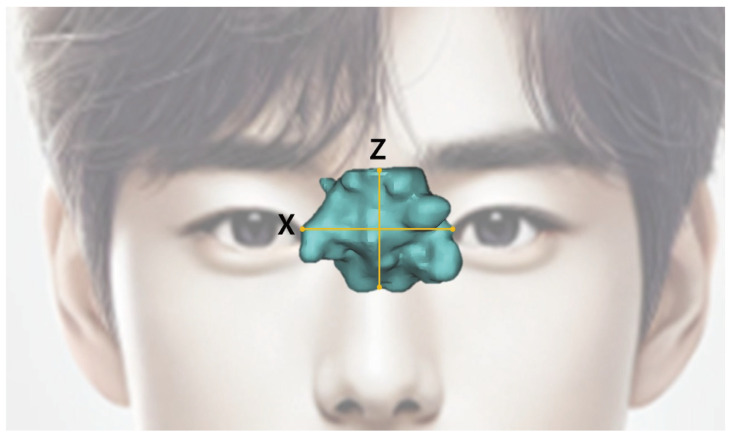
Sphenoid sinus measurement items by coronal view. (X) X-width: The maximum width in the coronal view. (Z) Z-width: The maximum height in the coronal view.

**Figure 6:**
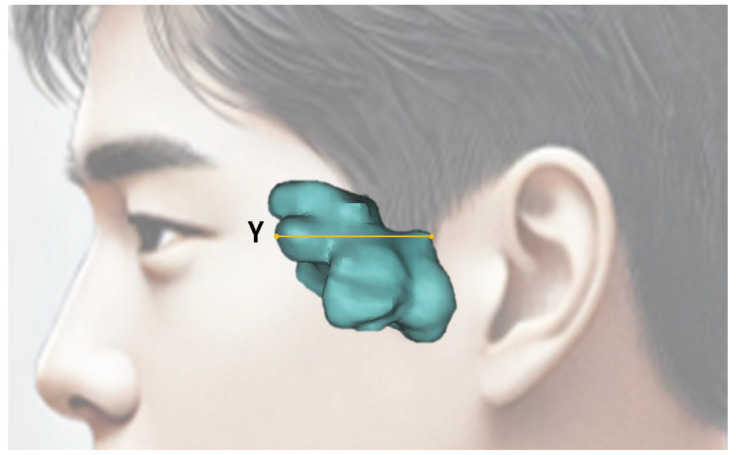
Sphenoid sinus measurement items by sagittal view. (Y) Y-width: The maximum width in the sagittal view.

The measurement parameters of the sphenoid sinus are presented in Table 1 below (Figure 5 and Figure 6).

#### 2.2.6. Critical Appraisal of Research Methodology

Despite the statistical justification of the sample size, it is acknowledged that this sample may have limitations in generalizability to larger populations, such as the national demographic. Therefore, this study is positioned as an exploratory investigation to establish baseline data. Participants underwent CBCT imaging for clinical purposes unrelated to this study, including dental implant planning, orthodontic evaluation, and assessment of impacted teeth. These clinical indications were recorded to evaluate their potential influence on the variability of sphenoid sinus size and morphology. By considering these factors, the study adopted a more nuanced approach to data interpretation. The segmentation process for image analysis was performed using Mimics software, which required manual corrections in certain cases, potentially introducing bias. To address this, independent cross-validation was conducted, and any discrepancies were resolved by consensus. Standardized HU thresholds and multi-view validation strategies were also applied to enhance segmentation accuracy and minimize errors. The Cronbach’s α value reported in this study was 0.709, indicating moderate reliability and raising concerns regarding measurement consistency. To account for this limitation, this study incorporated standardized imaging protocols and cross-validation strategies into the research design. Future studies are encouraged to utilize advanced imaging techniques to improve reliability and reduce variability. Despite these limitations, this study integrated various compensatory elements in its design to ensure robust findings and provide foundational data for subsequent research.

#### 2.2.7. Statistical Analysis

The measurement items were analyzed using the SPSS program (version 23.0, IBM Corporation, Armonk, NY, USA). Statistical methods included *t*-test for sex-based analysis of sphenoid sinus size, and linear regression analysis was performed to evaluate whether sphenoid sinus volume affected X-, Y-, and Z-widths. All statistical analyses were conducted with a 95% confidence interval, and the significance level was set at 0.05.

## 3. Results

### 3.1. Analysis of Sex Differences in Sphenoid Sinus Size

The results of the analysis of sex differences in sphenoid sinus size are shown in Table 2. The volume of the sphenoid sinus was 13,517.7 mm^3^ in females and 16,957.9 mm^3^ in males, showing a significant difference (*p* < 0.05). The X-width was 44.4 mm in females and 50.7 mm in males (*p* < 0.001), the Y-width was 34.5 mm in females and 40.5 mm in males (*p* < 0.001), and the Z-width was 30.3 mm in females and 32.2 mm in males (*p* < 0.001). These results indicate that the sphenoid sinus size is generally larger in males compared to females.

### 3.2. Analysis of the Effect of X-, Y-, and Z-Widths on Volume

To determine whether the X-, Y-, and Z-widths affect the overall volume of the sphenoid sinus, a simple linear regression analysis was performed. The results are shown in Table 3. The model was found to be suitable, with F = 155.048 (*p* < 0.05) and R^2^ = 0.800, indicating that the model explained 80% of the variance. The X-width had a β value of 0.029 and did not significantly affect the volume (*p* > 0.05). However, the Y-width (β = 0.192) and Z-width (β = 0.787) significantly influenced the volume (*p* < 0.001).

## 4. Discussion

The sphenoid sinus is an air-filled space located deep within the skull, surrounded by various structures such as the optic nerve, pituitary gland, and others [8]. Therefore, research is being conducted to prevent complications during sphenoid sinus surgery in clinical settings [9]. In the study by Unal et al. [9], it was reported that the anatomical variations of the sphenoid sinus are diverse, necessitating caution during surgery. Thus, the size and shape of the sphenoid sinus are clinically significant.

In forensic science, the sphenoid sinus is considered an important structure for personal identification because it is located deep within the skull and less susceptible to external damage. Uthman et al. [10] reported that morphological analysis of the sphenoid sinus can assist in personal identification, a finding echoed in the study by Deloire et al. [11]. As such, research on the sphenoid sinus is being conducted across various fields.

This study was conducted based on an exploratory study with the following objectives. The primary aim is to analyze the three-dimensional morphological differences of the sphenoid sinus according to sex in Korean adults aged 20–29 years. The sphenoid sinus, located deep within the skull, is relatively protected from external damage and has unique anatomical characteristics, which is why the 3D specialized software, Mimics, was used. Additionally, this study visualized and measured the sphenoid sinus using CBCT data from 120 Korean adults. The research findings were verified through two hypotheses. The hypotheses are as follows: First, “There is no significant difference in the size of the sphenoid sinus between male and female subjects”. Second, “The overall volume of the sphenoid sinus is not influenced by the dimensions measured along the Width from coronal view (X), Width from sagittal view (Y), and Height from coronal view (Z) axes”.

The first hypothesis states, “There is no significant difference in the size of the sphenoid sinus between male and female subjects”. The results of this study demonstrated that the sphenoid sinus was generally larger in males than in females, with statistically significant differences. Therefore, this hypothesis appears to be rejected based on the findings of this study. Similar results were found in the study by Ramos et al. [12]. A comparable study on sex differences of the maxillary sinus also showed that males had significantly larger sizes [2,13]. This difference is likely due to the fact that males tend to have larger skeletal structures than females. In the study by Milella et al. [14], it was reported that males have larger skulls than females. Therefore, the larger size of the sphenoid sinus in males may be attributed to hormonal differences that occur during growth, particularly the influence of testosterone on bone development and the size of air-containing spaces. Additionally, considering the overall structural dimensions of the skull and its relationship with the paranasal sinuses, these anatomical differences are likely not isolated traits but rather part of a broader cranial formation pattern. Future studies should quantitatively analyze these factors to provide a deeper understanding of the sex differences in sphenoid sinus size.

The second hypothesis states, “The overall volume of the sphenoid sinus is not influenced by the dimensions measured along the Width from coronal view (X), Width from sagittal view (Y), and Height from coronal view (Z) axes”. In this study, we examined whether the X-width, Y-width, and Z-width affect the volume. The results indicated that the Y-width and Z-width significantly influenced the volume. Therefore, this hypothesis appears to be rejected based on the findings of this study. According to previous research, the sphenoid sinus expands anteriorly and posteriorly in a parallel manner until the age of 10, and after the age of 15, it can expand in all directions [15]. Additionally, Jaworek-Troć et al. [16] reported that the sphenoid sinus expands anteriorly due to anatomical variations. Previous studies have indicated that variations in the sphenoid sinus can be influenced by factors such as genetics, trauma, hyperpneumatization, race, and environmental factors [17]. Therefore, further research on the anatomical variations of the sphenoid sinus across different age groups and races is needed.

This study conducted a three-dimensional analysis of the sphenoid sinus size and its differences by sex, identifying the anatomical characteristics of the sphenoid sinus in Korean adults. The findings provide practical applications in clinical and forensic practices, particularly in the following areas. First, in the clinical field, the differences in sphenoid sinus size by sex offer valuable information for surgical planning and diagnosis. The sphenoid sinus is adjacent to critical anatomical structures such as the orbit, optic nerve, and pituitary gland, making an accurate understanding of its size and morphology essential during endoscopic sinus surgery or skull base surgery. The finding that male patients tend to have larger sphenoid sinuses can serve as a useful guide for preoperative assessment of surgical approaches and risk factors. Furthermore, the CBCT-based 3D modeling technique used in this study allows precise evaluation of lesion extent in the sphenoid sinus and contributes to developing personalized treatment plans. Second, in forensic science, the differences in sphenoid sinus size by sex can play a significant role in personal identification. In cases where severe trauma or DNA sample degradation occurs, the size and morphology of the sphenoid sinus can be utilized to identify sex with relatively high accuracy. Notably, the unique anatomical characteristics of the sphenoid sinus make it an effective tool for comparing ante-mortem and post-mortem radiographic images to confirm an individual’s identity. These findings are expected to enhance the precision of forensic identification.

This study utilized the 3D modeling software Mimics to compare and analyze the size of the sphenoid sinus between sexes in Korean adults. Mimics is a powerful tool that converts 2D medical images, such as those from CT and MRI, into 3D models, making it particularly valuable in medical and forensic applications for precise anatomical modeling. Numerous studies have confirmed the measurement accuracy of Mimics, with high accuracy in 3D CT-based craniofacial and upper airway analysis. According to previous research, a comparative study between Mimics 17.0 and In Vesalius 3.0 software found no significant differences in measurements based on key anatomical landmarks, demonstrating that both programs are reliable [24]. Therefore, the morphology and volume of the sphenoid sinus in this study are considered to have been visualized with high accuracy.

This study analyzed sex-based differences in the sphenoid sinus to explore clinical and forensic applications; however, several limitations must be addressed. First, Exclusion of Genetic and Environmental Factors: Although sphenoid sinus size and morphology are likely influenced by genetic factors (e.g., familial traits, racial characteristics) and environmental factors (e.g., smoking, chronic sinusitis, air pollution), these variables were not included in the analysis. This omission limits the accuracy of result interpretation and hinders a comprehensive understanding of the factors affecting sphenoid sinus size. Second, Restricted Sample Characteristics: This study focused on Koreans aged 20–29 years, limiting the generalizability of findings to other age groups or ethnic populations. As sphenoid sinus development varies by age and ethnicity, future studies should include a broader range of demographic groups. Third, Cross-sectional Study Design: The cross-sectional nature of the study did not allow for the analysis of changes in sphenoid sinus size over time. Longitudinal studies are necessary to investigate the effects of growth, maturation, and aging on sphenoid sinus morphology. Fourth, Lack of Consideration for Medical and Lifestyle Histories: This study did not account for participants’ lifestyle factors or medical histories, such as smoking, alcohol consumption, or chronic illnesses, which may influence sphenoid sinus size. The absence of these variables could introduce potential confounding factors into the findings. Fifth, Absence of Individual Sphenoid Sinus Analysis: The analysis was conducted on the sphenoid sinuses as a whole rather than evaluating each side individually. This approach does not reflect potential asymmetry or individual-specific characteristics, limiting detailed interpretation. Sixth, Limited Reference to Previous Studies: Existing studies have predominantly focused on Western populations, and this study, centered on Koreans, does not sufficiently address inter-ethnic and regional differences. This limitation affects the global applicability of the results.

Despite these limitations, this study contributes valuable baseline data on the sex differences in the sphenoid sinus size among young Korean adults. The findings have implications for both clinical practice and forensic science, as they enhance our understanding of the anatomical variations of the sphenoid sinus and provide insight into how these variations can be used for sex identification.

## 5. Conclusions

This study aimed to investigate sex differences in the size of the sphenoid sinus among Koreans. Based on this, an exploratory study was conducted. The results showed that males had larger sphenoid sinuses than females, consistent with the findings of previous studies. Furthermore, the overall volume of the sphenoid sinus was found to be influenced by the Z-width (height in the coronal view) and the Y-width (width in the sagittal view), aligning with previous research on sinus expansion. Therefore, this study complements the limitations of previous research that primarily focused on Western populations and contributes to understanding the morphological characteristics of the sphenoid sinus in East Asian populations, including Koreans. These findings provide essential baseline data for enhancing the clinical diagnosis, particularly in planning surgeries involving the inner skull, and for improving the forensic identification process. However, further research is needed to include a wider range of subjects and to analyze the anatomical characteristics of the sphenoid sinus more precisely.

## Figures and Tables

**Table 1:** Sphenoid sinus measurement items.

Parameter	Definition
Volume	Total sphenoid sinus volume
X-width	Width from coronal view
Y-width	Width from sagittal view
Z-width	Height from coronal view

**Table 2:** Analysis of sphenoid dimensions based on sex.

Dimension	Sex	N	Mean (SD)	F	t	*p*
Volume	male	60	13,517.7 (4765.3)	4.628	−3.235	0.002
	female	60	16,957.9 (6718.8)		−3.235	0.002
X-width	male	60	44.4 (5.5)	46.131	−4.380	0.000 *
	female	60	50.7 (9.8)		−4.380	0.000 *
Y-width	male	60	34.5 (3.2)	18.297	−7.716	0.000 *
	female	60	40.5 (5.0)		−7.716	0.000 *
Z-width	male	60	30.3 (4.4)	0.741	−2.124	0.036
	female	60	32.2 (5.4)		−2.124	0.036

Data are mean (standard deviation values); *p*-values were obtained by *t*-test (* *p* < 0.001).

**Table 3:** Analysis of the effect of X-, Y-, and Z-widths on volume.

Measurements	B	SE	Β	t(p)	f(p)	R^2^
Constant	−24,033.671	2083.719		−11.534	155.048	0.800
X-width	20.572	38.557	0.029	0.534		
Y-width	225.053	57.375	0.192	0.192 **		
Z-width	954.717	60.559	0.787	0.787 **		

*p*-values were obtained by simple linear regression; ** *p* < 0.01.

## Data Availability

Original data are available upon request to the corresponding author.
